# Hybrid Supramolecular and Colloidal Hydrogels that Bridge Multiple Length Scales**

**DOI:** 10.1002/anie.201410570

**Published:** 2015-03-13

**Authors:** Emma-Rose Janeček, Jason R McKee, Cindy S Y Tan, Antti Nykänen, Marjo Kettunen, Janne Laine, Olli Ikkala, Oren A Scherman

**Affiliations:** Melville Laboratory for Polymer Synthesis, Department of ChemistryUniversity of Cambridge, Lensfield Road, Cambridge CB21EW (UK); Molecular Materials, Department of Applied Physics, Aalto University (previously Helsinki University of Technology)P.O. Box 15100, FIN-00076, Espoo (Finland); Department of Forest Products Technology, School of Chemical Technology, Aalto UniversityP.O. Box 16300, FIN-00076 Aalto (Finland)

**Keywords:** hydrogels, nanocellulose, nanocomposites, supramolecular chemistry

## Abstract

Hybrid nanocomposites were constructed based on colloidal nanofibrillar hydrogels with interpenetrating supramolecular hydrogels, displaying enhanced rheological yield strain and a synergistic improvement in storage modulus. The supramolecular hydrogel consists of naphthyl-functionalized hydroxyethyl cellulose and a cationic polystyrene derivative decorated with methylviologen moieties, physically cross-linked with cucurbit[8]uril macrocyclic hosts. Fast exchange kinetics within the supramolecular system are enabled by reversible cross-linking through the binding of the naphthyl and viologen guests. The colloidal hydrogel consists of nanofibrillated cellulose that combines a mechanically strong nanofiber skeleton with a lateral fibrillar diameter of a few nanometers. The two networks interact through hydroxyethyl cellulose adsorption to the nanofibrillated cellulose surfaces. This work shows methods to bridge the length scales of molecular and colloidal hybrid hydrogels, resulting in synergy between reinforcement and dynamics.

Recently, natural structural materials have inspired synthetic solvent-based and solid nanocomposite architectures.[1–6] Natural structural materials demonstrate a combined strength, stiffness, and toughness that are rarely achieved by synthetic materials, through bridging length scales and combining competing properties. Silk, animal bone, nacre, and plant fibers are all formed from self-assembled hierarchical structures across multiple length scales. Therein, hard reinforcing nanoscale domains are bound together by soft energy-dissipating molecular networks with sacrificial bonds, working together in synergy. Because of the highly complex structures, the synthetic production of related bioinspired networks and gels has proven to be challenging. However, some biomimetic materials exist, wherein “hard” colloidal reinforcement have been utilized to increase the strength and stiffness, in some cases even the toughness.[7–11]

One of the main strategies used by nature is the combination of colloidal-length-scale reinforcement with supramolecular bonds on a molecular level, thus allowing rapid dissociation and reformation.[12] Thus prepared nanocomposites that incorporate molecular-scale networks with rapid supramolecular exchange kinetics in addition to colloidal reinforcement were explored herein. Engineering of sacrificial bonds into solid cellulose nanocrystal nanocomposites using supramolecular construction principles to achieve engineered fracture energy dissipation was recently demonstrated.[13] Moreover, dynamic supramolecular bonds have been particularly attractive for self-healing supramolecular rubbers.[14]

The present study focuses on the combination of colloidal and molecular length scales in hydrogels to engineer a dynamic supramolecular sacrificial network that promotes stiffness and connectivity in nanofibrillated cellulose (NFC) colloidal hydrogels. Pure NFC forms hydrogels, in which the skeleton is formed by native cellulose nanofibers with lateral diameters of nanometers and lengths up to several micrometers.[15] NFC has been used for a wide range of emerging applications, such as high-strength nanomaterials and biomedical scaffolds.[16–20] NFC is extracted from plant cell walls and has high elastic moduli (26–36 GPa) and tensile strength in the GPa range.[21–23] Dispersed NFC forms stiff hydrogels that are thought to be mediated by fibril–fibril entanglement facilitated by hydrogen bonding.[15] However, the stiff crystalline domains of the nanofibrils allow little yielding and stress dissipation, hence the hydrogels prepared from NFC tend to be brittle. NFC hydrogels demonstrate shear thinning below 100 % strain. Furthermore, there are challenges associated with achieving a homogeneous distribution in the aqueous medium. Within NFC hydrogels, denser “floc-like” regions typically form, separated by less dense “weak links” (Figure 1 b, Figure 5 b).[24,25] A lack of dynamics within NFC cross-links coupled with separated material domains led us to the use of dynamic supramolecular linkages to enhance stiffness and yield strain, thus targeting significant challenges associated with the utilization of NFC hydrogels. NFC hydrogels are therefore our reference material herein.

**Figure 1:**
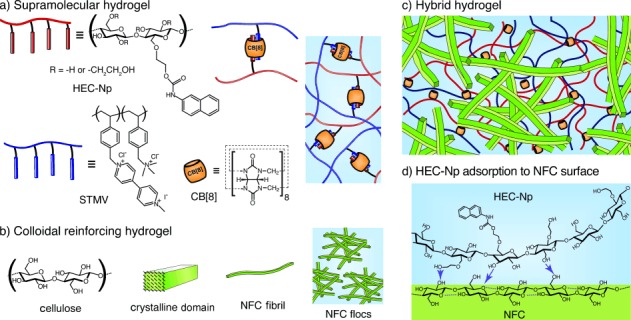
a) Supramolecular hydrogel consisting of HEC-Np, STMV, and the CB[8] host motif capable of binding the first guest naphthyl and the second guest viologen highly dynamically. b) Colloidal reinforcing nanofibrillated cellulose, also showing the denser and less dense network regimes (see Figure 5). c) Interpenetrating hybrid hydrogel consisting of the molecular-level supramolecular and colloidal-level NFC hydrogel. Because of its rigid nanofibers and short aspect ratio, network aggregates are formed (flocs). d) Surface adsorption of HEC-Np onto the NFC surface. Possible hydrogen bonding is schematically shown.

**Figure 5:**
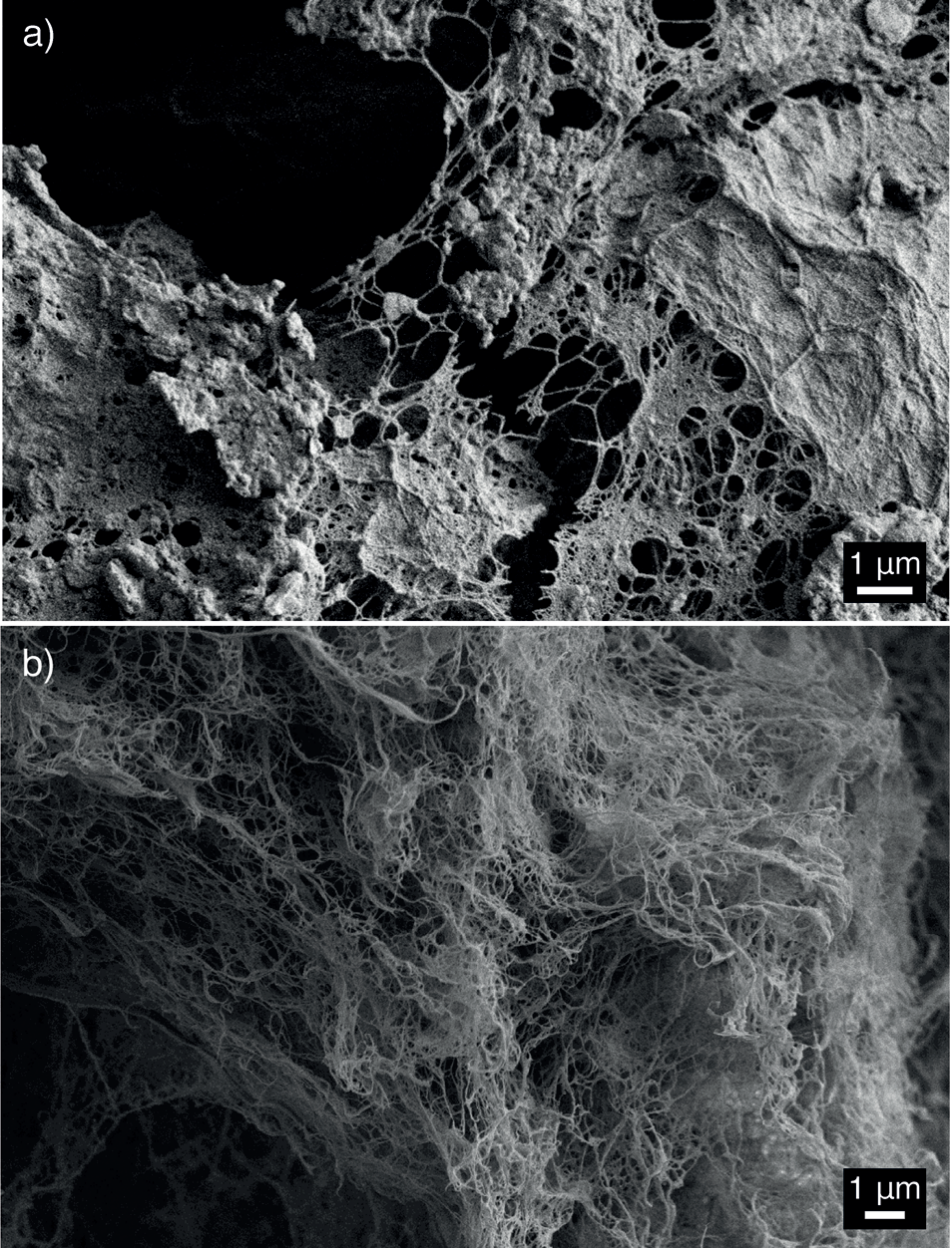
a) SEM image of the nanocomposite hydrogels with NFC loading (1 wt. %). Note the supramolecular polymer between the fibrils. b) SEM image of the pristine NFC (1 wt. %). Note the highly fibrillar structure.

Supramolecular interactions embedded within hydrogels have allowed for stimuli responsivity, controlled unzipping, and supramolecular cross-link reformations without immediate failure of the entire network.[26,27] Specifically, the host–guest interactions of cucurbit[8]uril (CB[8]) display reversible complexation at rates approaching the diffusion limit.[28] CB[8] can form 1:1:1 heteroternary complexes with two guest motifs, still allowing very high binding constants in spite of the dynamic nature. In this study, first-guest methylviologen (MV) and second-guest naphthyl (Np) functional polymers were used. Accordingly, the dynamic CB[8] network was formed using naphthyl-functionalized hydroxyethyl cellulose (HEC-Np) and a viologen functional styrene derivative (STMV; Figure 1 a). Related polymeric constituents have previously been shown to be compatible with each other.[29] Adsorbing functional polysaccharides, such as HEC, to a nanocellulose surface has also been demonstrated to be an effective method to form physically cross-linked nanocellulose networks.[30–32] Favorable interfacial interactions improve the rheological properties of nanocomposite hydrogels.[29] Hence, an interconnected system was foreseen whereby the HEC-Np “glued” the molecular-scale supramolecular network to the NFC colloidal scale network (Figure 1 c).

In more detail, the architecture comprised of a colloidal NFC network enhanced by association with a dynamic supramolecular network (Figure 1 c). A CB[8]-mediated supramolecular hydrogel, which displayed the favorable stiffness yet minimal solids content of fixed amounts of HEC-Np (5 mol % Np loading), PSTMV (10 mol % MV loading), and CB[8],[33] was investigated in combination with variable loading of the NFC hydrogel. A fixed composition of the supramolecular hydrogel was used, incorporating 0.5 wt. % HEC-Np, 0.15 wt. % STMV, and 0.1 wt. % CB[8], which corresponds to nominal equimolar amounts of both naphthyl, viologen and CB[8]. To this fixed composition, the NFC colloidal hydrogel was added (0–1.5 wt. %). For reference, CB[8] was removed or replaced with CB[7] to show the essential role of the dynamic supramolecular interactions. Note that CB[7] does not allow simultaneous binding of viologens and naphthyls.

By combining a variable amount of NFC (0–1.5 wt. %) with fixed amounts of STMV (0.15 wt. %) and CB[8] (0.1 wt. %), no apparent increase in the storage modulus was observed. Good compatibility was inferred as no apparent aggregation or phase separation occurred. Next, a fixed amount of HEC-Np (0.5 wt. %) was added to form the heteroternary complex and to attach the ensuing supramolecular hydrogel to the NFC hydrogel through adsorbing the HEC to the NFC surface. This resulted in synergistic enhancement of the low-strain elastic modulus as the two networks were bound together (Figure 2 a). The storage modulus data from the NFC colloidal hydrogel alone at varying weight content shows good agreement with a published model describing the rheological properties of NFC hydrogels.[34] In order to carry out this fitting, a value of fiber Young’s modulus of 31 GPa was used based on published data.[23] A void volume fraction of 0.434 was calculated for the pure NFC colloidal hydrogel used here according to the fit shown in Figure 2. The stiffening of the hybrid gel was clearly mediated by the heteroternary CB[8] complex, as in its absence, rheological properties of the gel were only slightly increased relative to pristine NFC at the same concentration (Figure 2 a, and Figure S1 in the Supporting Information), as shown by the low-strain modulus values. This result demonstrated that the specific CB[8]-dependent interactions were essential for the promoted rheological properties. In the absence of CB[8], or using smaller host homologue CB[7], which does not form the ternary complex, only a slight increase in the storage modulus was observed.

**Figure 2:**
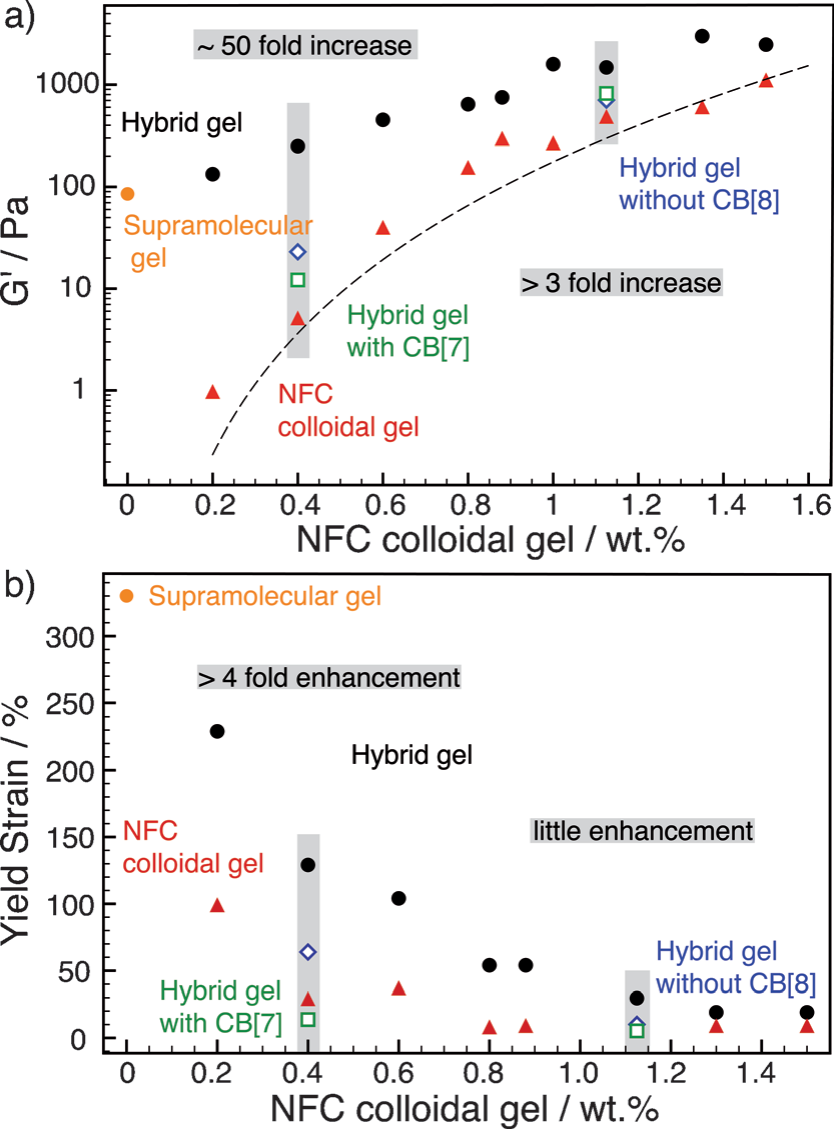
Rheology of the hybrid supramolecular and NFC colloidal hydrogel, as the concentration of the NFC colloidal gel is increased from 0 to 1.5 wt. %, while the supramolecular hydrogel composition is kept fixed. a) Storage modulus determined in the linear regime (0.1–1 % strain) at 10 rad/s. The hybrid gel (black circles) has higher *G*′ values than either the corresponding NFC reference (red triangles) or the supramolecular gel (orange circle). Fitting of the pristine NFC data was done according to a published model[34] (dashed line). b) Yield strain determined at 10 rad/s. For comparison, selected reference materials are also shown: hybrid hydrogel without CB[8] (blue diamonds), and hybrid hydrogel where CB[8] has been replaced by CB[7] (green squares).

As well as increasing the low-strain *G*′, the addition of the CB[8] network to the NFC network resulted in increased yield strain (Figure 2 b). It is normally observed that an increase in the covalent cross-linking density within a polymeric hydrogel will lead to an increase in *G*′ and a decrease in yield strain.[35] This is on account of the shorter distance between cross-links and hence a reduction in polymer elongation before pinning by a cross-link. Here, the addition of the dynamic supramolecular hydrogel bonding the NFC colloidal hydrogel domains together—resulted in an increase in *G*′. Furthermore, CB[8] binding led to more dynamic interactions, allowing reconfiguration upon deformation, which resulted in enhanced elasticity and yield strain. However, this effect was less pronounced at high NFC weight content, at which the rheological properties of the hydrogel were dominated by the NFC.

In order to further investigate the hybrid hydrogel with different NFC colloidal gel loadings, but constant total solid content, the ratio between the two gel networks was varied while the total solid concentration was held constant at 1.15 wt. %. As shown in Figure 3, there was no significant increase in *G*′ over the compositions investigated, however, the yield strain was increased by more than an order of magnitude, as the relative amount of the supramolecular hydrogel fraction increased. This observation can be explained by reconfigurations of supramolecular bonds, hence allowing the hybrid hydrogel to sustain greater shear strain before breakage of the overall nanocomposite. The change in yield strain with increasing supramolecular gel content falls between the curves calculated according to the rule of mixtures or the inverse rule of mixtures. This is as expected for a composite material with no alignment of fibers. Adherence to the rule of mixtures indicates interaction between the two hydrogel networks supporting the hypothesized mechanism of rheological enhancement.

**Figure 3:**
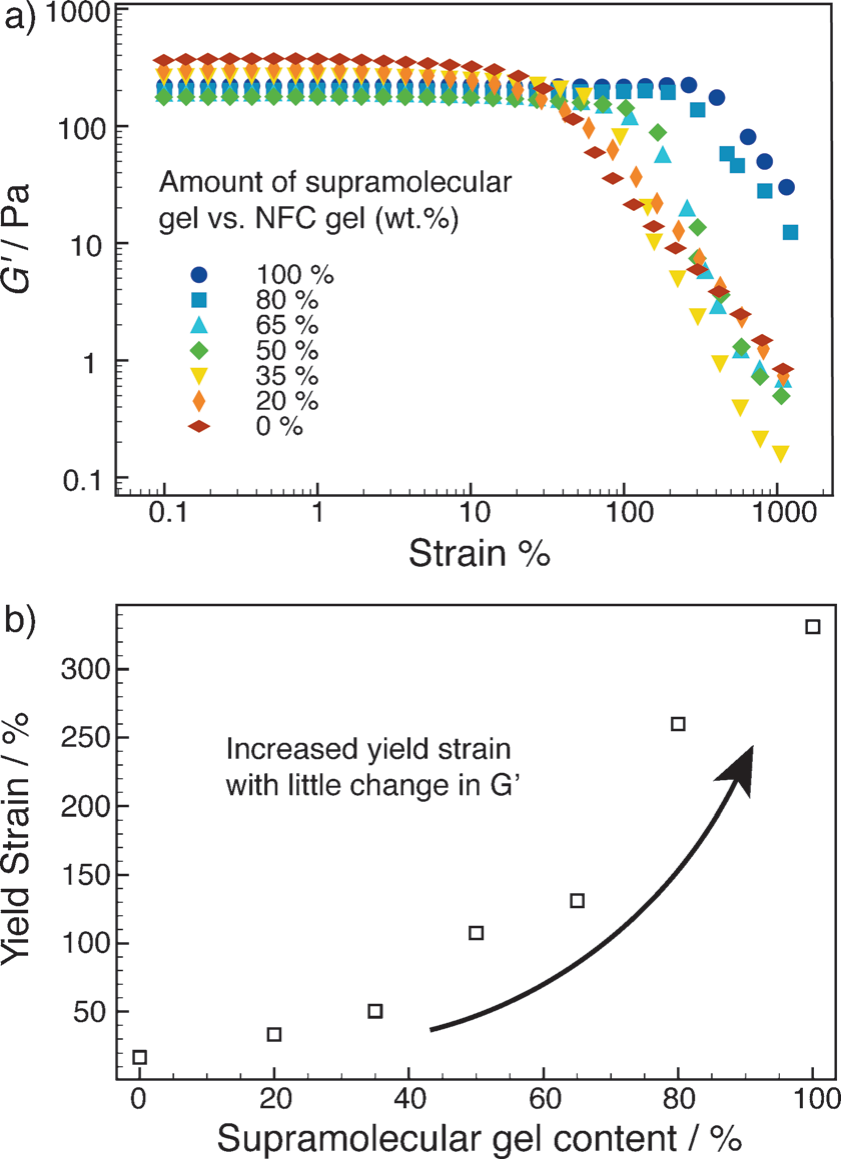
Rheology of the hybrid supramolecular and NFC colloidal hydrogel upon keeping the total amount of solids constant at 1.15 wt. % with changing the weight content of the supramolecular gel vs. the NFC colloidal gel. a) Storage modulus. b) Yield strain.

Strain amplitude sweeps performed on pristine NFC colloidal hydrogels showed that once strained beyond the gel-to-sol transition and retested, the low-strain *G*′ was essentially unchanged (Figure S2); however, the yield strain was reduced with repeated gel-to-sol transitions (Figure 4 a). This observation suggests that the low strain *G*′ was dependent on reversible entanglement and interactions between the NFC fibrils and hence was able to recover fairly quickly. However, the yield strain was likely dependent on the strongest entanglements within the NFC network, which were broken by each repeat of the sol-to-gel transition and hence the yield strain was decreased with each repeat (Figure 4 a). The hybrid supramolecular and NFC colloidal hydrogels, with the same supramolecular gel content as in Figure 2, however, demonstrated different behavior when repeatedly subjected to amplitude sweeps. Between repeat 1 and 2, there was a dramatically decreased yield strain, again indicating breakage of the strong, yet brittle NFC network. Yet, with subsequent repeats, the decrease in the yield strain was less pronounced. It is therefore proposed that not only was the hybrid hydrogel able to dissipate mechanical energy within the strained system, it was also able to mediate partial repair through rapid healing of the CB[8]-based supramolecular gel network. The CB[8]-based supramolecular hydrogel alone is known to self-heal rapidly and completely upon shearing, hence this hydrogel cannot account for the decreased yield strain.[33]

**Figure 4:**
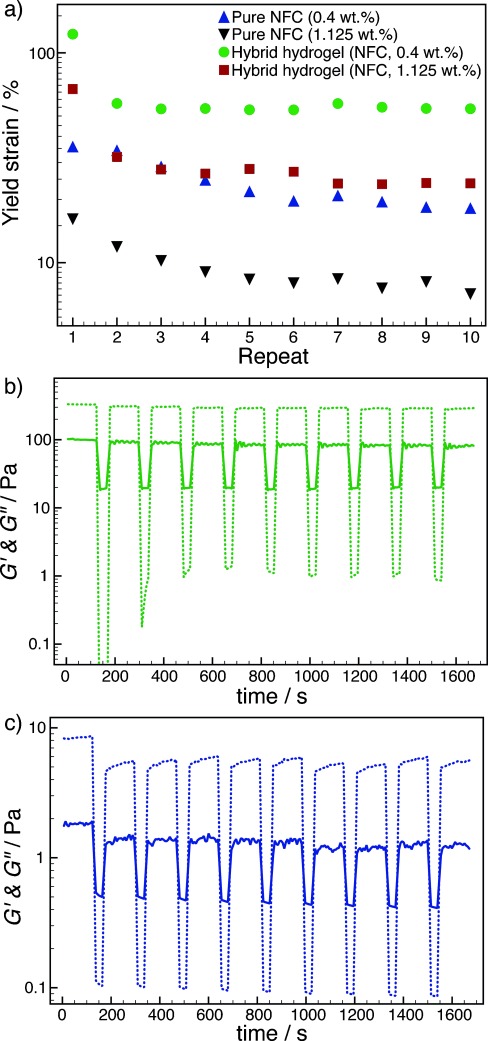
a) Sequential yield strain after breaking down the hydrogel network and allowing it to reform in repeated amplitude-sweep rheological experiments (▪) hybrid hydrogel with 1.125 wt. % NFC loading; (▾) reference pure NFC colloidal hydrogel 1.125 wt. %; (•) hybrid hydrogel with 0.4 wt. % NFC loading; (▴) reference pure NFC colloidal hydrogel 0.4 wt. %. Yield strain calculated from the intercept of the mean *G*′ over the first decade of strain % with the *G*′ slope after yielding. b, c) step-strain rheological experiments. b) Hybrid supramolecular and NFC colloidal hydrogels with 0.4 wt. % NFC loading. c) Pure colloidal NFC hydrogel with 0.4 wt. % loading. *G*′ (dashed lines) and *G*′′ (solid lines) measured over steps of 0.1 % and 600 % oscillatory strain.

Step-strain experiments (Figure 4 b and c) illustrate this CB[8]-mediated recovery. First, a 0.4 wt. % pristine NFC colloidal hydrogel was subjected to 0.1 % strain, well below the gel-to-sol transition, and then to 600 % strain, well above the gel-to-sol transitions, and then retested at 0.1 % strain. This cycle was then repeated to include ten low strain periods. It was found that although the initial *G*′ was not fully recovered after the first cycle, a gel was again present at low strain, and additional cycles resulted in minimal decrease in the *G*′. A significant time dependence of the *G*′ was however noted, as during the 120 s at low strain, *G*′ clearly increases. Note the curvature of the *G*′ plot in all low strain periods after the first repeat (Figure 4 c). This suggests that re-entanglement of the NFC hydrogel was able to occur at room temperature under low-shear conditions. This experiment was repeated for hybrid hydrogels at higher NFC concentration and a similar trend was observed (Figure S3), however the 600 % strain was no longer within the linear viscoelastic regime of the material. When the step–strain experiments were carried out on the hybrid supramolecular and colloidal hydrogels with 0.4 wt. % NFC loading, an improved recovery in *G*′ was achieved after the initial gel-to-sol transition and the time dependence on the *G*′ recovery was less pronounced than for pristine NFC (Figure 4 c). This result suggests that the self-healing of the CB[8]-based supramolecular gel network, thought to be in the order of the diffusion limit, was able to mediate recovery of the hybrid gel. Self-healing of the weak CB[8]-based supramolecular gel network was therefore able to mediate healing of the NFC network, as the strength of the gel recovered was vastly above that of the CB[8]-based supramolecular gel network alone. Rapid healing of the supramolecular hydrogel likely heals the interconnections of the dense regimes of NFC colloidal gels.

Finally, scanning electron microscopy (SEM) images support the schematic description of the network (Figure 1 c and a), as the connections can clearly be seen between the NFC fibrils, whereas the pure NFC displays only a highly fibrillar structure (Figure 5 b). Therefore, SEM illustrates the ability of the supramolecular hydrogel to bridge between the denser “floc-like” domains of the NFC colloidal hydrogel, leading to a reinforced hybrid nanocomposite. It is clearly illustrated, therefore the ability of the two hydrogel networks to interpenetrate and interact with each other. Cryo-TEM, on the other hand, demonstrates that the fibrillar structure remained intact even after the addition of the supramolecular network. This further indicated that all the components were compatible (Figure S4).

In conclusion, a supramolecular hydrogel bridges the colloidal nanofibrillar NFC hydrogel domains, leading to significantly enhanced storage modulus values and improved maximum elastic yield values. This was promoted by the fast dissociation/association dynamics of the supramolecular CB[8]-based supramolecular cross-links in combination with the adsorption of hydroxyethyl cellulose component of the supramolecular hydrogel to the NFC nanofibers. The work shows biomimetic routes to combine components of different length scales and physical interactions to tune rheological properties.

## References

[1] Fratzl P, Weinkamer R (2007). Prog. Mater. Sci.

[2] Bhushan B (2009). Philos. Trans. R. Soc. London Ser. A.

[3] Espinosa HD, Rim JE, Barthelat F, Buehler MJ (2009). Prog. Mater. Sci.

[4] Fantner GE, Hassenkam T, Kindt JH, Weaver JC, Birkedal H, Pechenik L, Cutroni JA, Cidade GAG, Stucky GD, Morse DE, Hansma PK (2005). Nat. Mater.

[5] Keten S, Xu Z, Ihle B, Buehler MJ (2010). Nat. Mater.

[6] Meyers MA, Chen P-Y, Lin AY-M, Seki Y (2008). Prog. Mater. Sci.

[7] Tang Z, Kotov NA, Magonov S, Ozturk B (2003). Nat. Mater.

[8] Capadona JR, van den Berg O, Capadona LA, Schroeter M, Rowan SJ, Tyler DJ, Weder C (2007). Nat. Nanotechnol.

[9] Munch E, Launey ME, Alsem DH, Saiz E, Tomsia AP, Ritchie RO (2008). Science.

[10] Walther A, Bjurhager I, Malho JM, Pere J, Ruokolainen J, Berglund LA, Ikkala O (2010). Nano Lett.

[11] Bonderer LJ, Studart AR, Gauckler LJ (2008). Science.

[12] Fantner GE, Oroudjev E, Schitter G, Golde LS, Thurner P, Finch MM, Turner P, Gutsmann T, Morse DE, Hansma H, Hansma PK (2006). Biophys. J.

[13] McKee JR, Huokuna J, Martikainen L, Karesoja M, Nykänen A, Kontturi E, Tenhu H, Ruokolainen J, Ikkala O Angew. Chem. Int. Ed.

[14] Cordier P, Tournilhac F, Soulié-Ziakovic C, Leibler L (2008). Nature.

[15] Pääkkö M, Ankerfors M, Kosonen H, Nykänen A, Ahola S, Österberg M, Ruokolainen J, Laine J, Larsson PT, Ikkala O, Lindström T (2007). Biomacromolecules.

[16] Eichhorn SJ (2011). Soft Matter.

[17] Eichhorn SJ, Dufresne A, Aranguren M, Marcovich NE, Capadona JR, Rowan SJ, Weder C, Thielemans W, Roman M, Renneckar S, Gindl W, Veigel S, Keckes J, Yano H, Abe K, Nogi M, Nakagaito AN, Mangalam A, Simonsen J, Benight AS, Bismarck A, Berglund LA, Peijs T (2010). J. Mater. Sci.

[18] Siqueira G, Bras J, Dufresne A (2010). Polymer.

[19] Shanmuganathan K, Capadona JR, Rowan SJ, Weder C (2010). Prog. Polym. Sci.

[20] Missoum K, Belgacem M, Bras J (2013). Materials.

[21] Šturcová A, Davies GR, Eichhorn SJ (2005). Biomacromolecules.

[22] Saito T, Kuramae R, Wohlert J, Berglund LA, Isogai A (2013). Biomacromolecules.

[23] Tanpichai S, Quero F, Nogi M, Yano H, Young RJ, Lindstrom T, Sampson WW, Eichhorn SJ (2012). Biomacromolecules.

[24] Karppinen A, Saarinen T, Salmela J, Laukkanen A, Nuopponen M, Seppala J (2012). Cellulose.

[25] Bjorkman U (2005). Nord. Pulp Pap. Res. J.

[26] Guo M, Pitet LM, Wyss HM, Vos M, Dankers PYW, Meijer EW (2014). J. Am. Chem. Soc.

[27] Sun J-Y, Zhao X, Illeperuma WRK, Chaudhuri O, Oh KH, Mooney DJ, Vlassak JJ, Suo Z (2012). Nature.

[28] Appel EA, Forster RA, Koutsioubas A, Toprakcioglu C, Scherman OA Angew. Chem. Int. Ed.

[29] McKee JR, Appel EA, Seitsonen J, Kontturi E, Scherman OA, Ikkala O (2014). Adv. Funct. Mater.

[30] Laine J, Lindström T, Nordmark GG, Risinger G (2000). Nord. Pulp Pap. Res. J.

[31] Filpponen I, Kontturi E, Nummelin S, Rosilo H, Kolehmainen E, Ikkala O, Laine J (2012). Biomacromolecules.

[32] McKee JR, Hietala S, Seitsonen J, Laine J, Kontturi E, Ikkala O (2014). ACS Macro Lett.

[33] Appel EA, Loh XJ, Jones ST, Biedermann F, Dreiss CA, Scherman OA (2012). J. Am. Chem. Soc.

[34] Hill RJ (2008). Biomacromolecules.

[35] Appel EA, Biedermann F, Rauwald U, Jones ST, Zayed JM, Scherman OA (2010). J. Am. Chem. Soc.

