# Supplementary material for: Hybrid Supramolecular and Colloidal Hydrogels that Bridge Multiple Length Scales
Source: Angew Chem Int Ed Engl. 2015 Mar 13;54(18):5383–8. doi: 10.1002/anie.201410570 (PMC4471571; doi:10.1002/anie.201410570)
Supplement: Supplementary file 1 [file anie0054-5383-sd1.pdf]

Angewandte Chemie
Eine Zeitschrift der Gesellschaft Deutscher Chemiker

Supporting Information

Hybrid Supramolecular and Colloidal Hydrogels that Bridge Multiple Length Scales**

Emma-Rose Janeček, Jason R. McKee, Cindy S. Y. Tan, Antti Nykänen, Marjo Kettunen, Janne Laine, Olli Ikkala, and Oren A. Scherman**

anie_201410570_sm_miscellaneous_information.pdf

Experimental

Chemicals were purchased from Sigma Aldrich Chemical Company (Dorset, UK) and used as received. CB[8][1] was synthesized according to the published procedures referenced. NFC was prepared using standard methods. A review of NFC preparation methods is referenced for further information.[2]

Instrumentation and materials

Rheology was carried out on a TA Instruments (Hertfordshire, UK) DHR-2 controlled stress hybrid rheometer. Frequency sweeps were carried out at a strain amplitude of 10%. Amplitude sweeps measured between 0.1% and 1000 % oscillation strain were carried out at an angular frequency of 10 rad/s. Step strain measurements were measured at 0.1% and 600% oscillatory strain with an angular frequency of 10 rad/s and the steps measured for 120 sec and 30 sec respectively. All measurements were carried out at with a peltier plate set to 20°C using a 40 mm parallel-plate geometry and analysed using TA Orchestrator software. ^{1}H NMR was carried out on a 400 MHz Avance III HD Smart Probe spectrometer. Chemical shifts measured relative to TMS as an external reference with any residual solvent protons as an additional internal reference.

SEM protocol

1 wt. % NFC gel with or without CB[8] network were deposited on copper (1 mm thickness) and flash-frozen in liquid propane and freeze-dried. The resulting areogels were sputter coated with

*To whom correspondence should be addressed

Au/Pd (2 min, 30 mA) and images using a Zeiss (Jena, Germany) FE-SEM with variable pressure at 1.5 keV.

Cryo-TEM protocol

Small drops of hydrogel were deposited into a Quantifoil hole carbon grid. The samples were the blotted five times in an environmental chamber of FEI (Hillsboro, OR, USA) Vitrobot at 100 % relative humidity and temperature of 22 °C and then vitrified in a mixture of liquid ethane and propane (-180 °C). Zero-loss imaging of vitrified samples was carried out with JEOL (Tokyo, Japan) JEM-3200FSC 300kV TEM operated at liquid nitrogen temperatures.

Synthetic methods

Synthesis of Styrene methyl viologen monomer (StMV)

Synthesis carried out according to previously published protocol.[3] Mono methyl bipyridene (1.21 g, 4.06 mmol) and 4-vinylbenzyl chloride (0.69 g, 0.63 ml, 4.5 mmol) were dissolved in acetonitrile (40 ml) and bubbled with nitrogen before heating to 60 °C overnight. The mixture was allowed to cool and the solid isolated by filtration to yield a red solid (1.16 g, 2.57 mmol, 63 %). ^{1}H NMR (D_2O, 400MHz): δ = 9.1 (d, J = 6.7 Hz, 2H), 8.9 (d, J = 6.7 Hz, 2H), 8.49 (d, J = 6.7 Hz, 2H), 8.44 (d, J = 6.7 Hz, 2H), 7.57 (d, J = 7.5 Hz, 2H), 7.42 (d, J = 7.3 Hz, 2H), 6.77 (dd, J = 17.9, 11 Hz, 1H), 5.87 (d, J = 11 Hz, 1H), 5.87 (s, 2H), 5.34 (d, J = 11 Hz, 1H), 4.44 (s, 3H).

Synthesis of PSTMV

PSTMV was synthesised according to previously published protocols.[4] Vinylbenzyl (trimethylammonium) chloride (4.65 g, 22 mmol), StMV (1,1 g, 2.44 mmol) and azobiscyanovaleric acid (33.6 mg, 0.12 mmol) were dissolved in H_2O:Ethanol (1:1 by volume to make a total of 32 ml). The solution was then bubbled with nitrogen for 30 min and then heated to 70 °C for 48 h. The material was then precipitated in diethyl ether and the THF before freeze drying from aqueous

solution to yield an orange solid (3.57 g). ^{1}H NMR (D_2O, 400MHz): δ = 9.01 (m, viologen), 8.48 (m, viologen), 7.21 (m, stryene), 6.6 (m), 4.37 (m), 2.91-0.80 (polymer back bone).

Synthesis of HEC-Np

HEC-Np was synthesised according to previously published protocols.[5] Hydroxyethyl cellulose (2 g, 1.3 million Mw) was dissolved in NMP (240 ml) and under nitrogen the mixture heated was to 120 °C overnight to yield a pale yellow clear solution. To the room temperature solution was added 2-naphthol isoocyanate (60.2 mg, 3.55 mmol) and 11 drop of dibutyl tin dilaurate and the solution stirred overnight. The solution was the precipitated from cooled acetone. The solid was isolated by filtration, then dissolved in H_2O at 40°C before dialysis against H_2O overnight before freeze drying to yield a white solid. ^{1}H NMR (D_2O, 400MHz): δ = 7.89-7.34 (m, nathphyl), 4.28-2.89 (polymer backbone).

Gel preparation, example procedure

The ratio of NFC to CB[8] gel was varied as indicated in the text and, hence, the solid content varied accordingly. The general preparation method was constant for all samples, an example is described here for a NFC/HEC-Np/PSTMV/CB[8] 0.4/0.5/0.15/0.1 wt.%/wt.%/wt.%/wt.% gel. The solid content of the aqueous NFC stock suspension was 2.25 wt.%. NFC stock suspension (178 mg) was added to a mixture of CB[8] (1 mg) and PSTMV (1.5 mg), diluted with MilliQ water (322 mg) and vortexed to dissolve the CB[8]. To this mixture was added aqueous HEC-Np (500 mg, 10 mg/mL). As soon as combined gelation started to occur. The gel was vortexed to achieve homogeneity of components and then centrifuged to removed any bubbles which may have formed. A pale amber coloured gel resulted.

When NFC only gels were formed a portion of the stock suspension was simple diluted with MilliQ water to achieve the required solid concentration to a total of 1 mL.

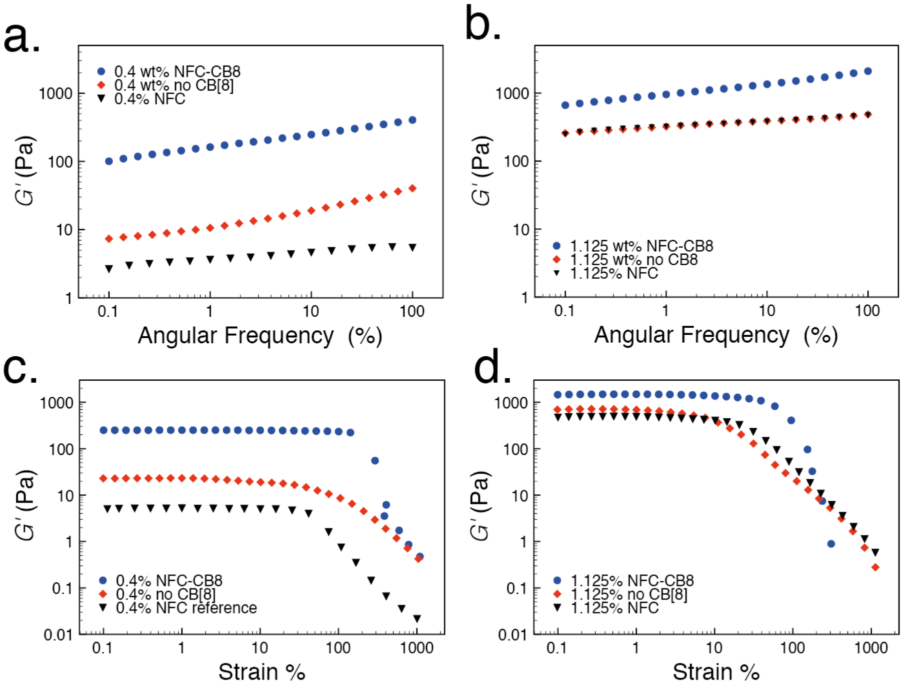

Figure S1: Characterization by dynamic oscillatory rheology: (blue circles) hybrid nanocomposite hydrogel with 0.4 or 1.125 wt.% NFC loading; (red diamonds) corresponding reference sample with all components except CB[8] host; and (black) corresponding NFC reference. a), b) Frequency sweeps as determined at 10 % strain for the samples that contain 0.4 and 1.125 wt.% NFC. c), d) Strain sweeps, as determined at 10 rad/s for the samples that contain 0.4 and 1.125 wt.% NFC samples.

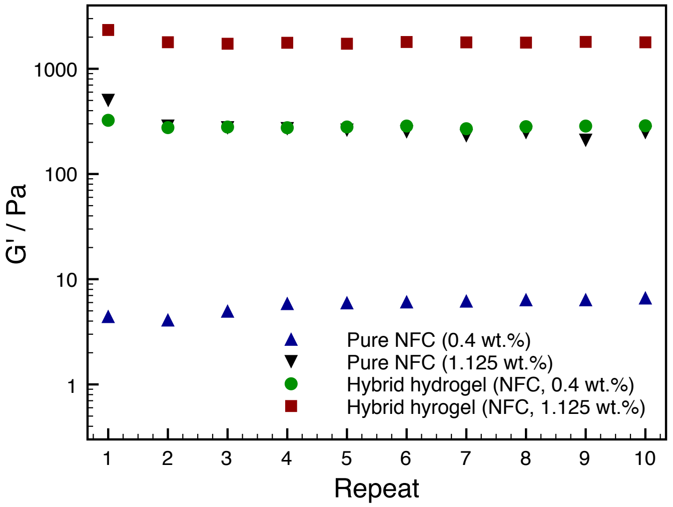

Figure S2: Variation in G' with repeated amplitude sweeps. (red squares) NFC/CB[8] gel 1.125/0.75 wt.%, (black downward triangles) NFC 1.125 wt.%, (green circles) NFC/CB[8] gel 0.4/0.75 wt.%, (blue upward triangles) NFC 0.4 wt.%. Yield strain calculated from the intercept of the mean G' over the first decade of strain % with the G' slope after yielding.

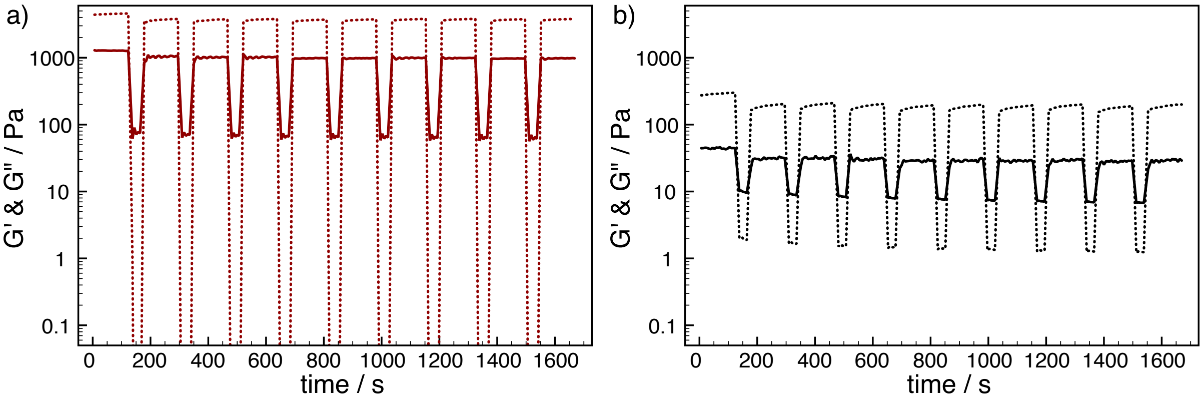

Figure S3: a) (red) NFC/CB[8] gel 1.125/0.75 wt.%, b) (black) NFC 1.125 wt.%. G' (solid lines) and G" (dashed lines) measured over steps of 0.1% and 600% oscillatory strain.

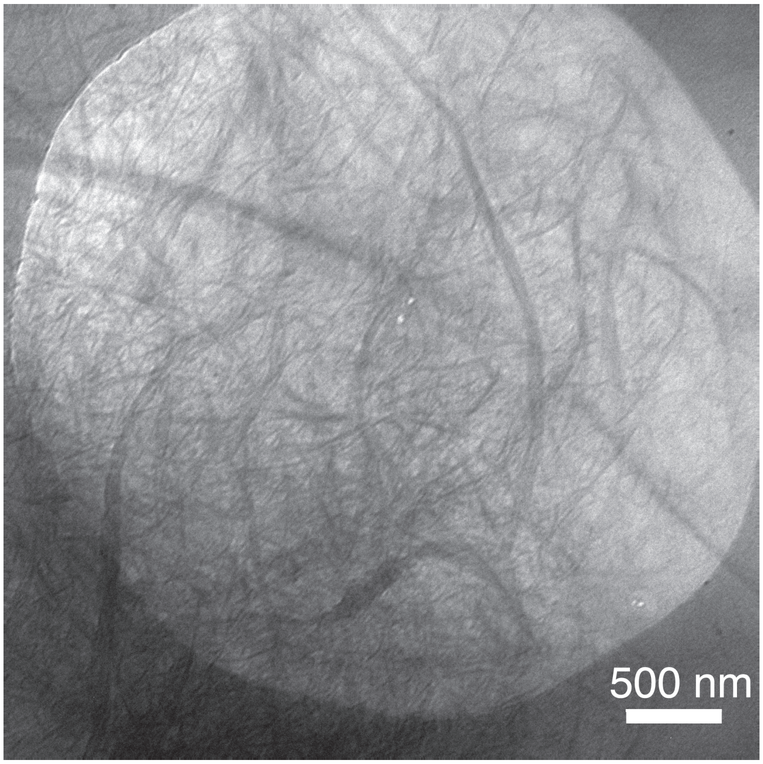

Figure S4: a) Cryo-TEM micrograph of NFC/CB[8] 1.00/0.75 wt.%.

References

[1] Kim, J.; Jung, I.-S.; Kim, S.-Y.; Lee, E.; Kang, J.-K.; Sakamoto, S.; Yamaguchi, K.; Kim, K. *J. Am. Chem. Soc.* **2000**, *122*, 540–541.

[2] Khalil, H. P. S. A.; Davoudpour, Y.; Islam, M. N.; Mustapha, A.; Sudesh, K.; Dungni, R.; Kawaid, M. *Carbohyd. Polym.* **2014**, *99*, 649–665.

[3] Ogoshi, T.; Masuda, K.; Yamagishi, T.-a.; Nakamoto, Y. *Macromolecules* **2009**, *42*, 8003–8005.

[4] Appel, E. A.; Biedermann, F.; Rauwald, U.; Jones, S. T.; Zayed, J. M.; Scherman, O. A. *J. Am. Chem. Soc.* **2010**, *132*, 14251–14260.

[5] Appel, E. A.; Loh, X. J.; Jones, S. T.; Biedermann, F.; Dreiss, C. A.; Scherman, O. A. *J. Am. Chem. Soc.* **2012**, *134*, 11767–11773.
